# Egocentric Fairness Perception: Emotional Reactions and Individual Differences in Overt Responses

**DOI:** 10.1371/journal.pone.0088432

**Published:** 2014-02-28

**Authors:** Benoit Bediou, Klaus R. Scherer

**Affiliations:** 1 Swiss Center for Affective Sciences, University of Geneva, Geneva, Switzerland; 2 Brain and Learning Lab, University of Geneva, Geneva, Switzerland; University of Queensland, Australia

## Abstract

Extensive research documents the existence of egocentric biases in the perception and application of justice norms. The origin of these biases remains poorly understood. We investigated both inter- and intra-individual differences in egocentric justice biases. Participants played an ultimatum game presumably with different anonymous players (simulated by a computer) in which they contributed differentially to the joint production of the initial endowment. We examined how contributions (low vs. high) affect proposers' offers and responders' acceptance decisions, as well as their fairness judgments and their emotional reactions to different types of offers (equal, equitable, unfair, and hyperfair). An egocentric bias in proposers' offers (indicating more flexible preferences) was found only in individualists and not in prosocials, suggesting differences in the motivations (or cognitions) underlying their choice of justice norms. Responders also showed egocentric biases in their judgments of fairness and in their emotional reactions to equal and equitable offers, but not in their acceptance decisions. Such dissociation might suggest that some form of emotion regulation occurred. Responders may evaluate offers on valence dimensions (e.g., goal conduciveness/outcome favorability and norm compatibility/justice) that are multiply interacting and potentially conflicting. The individual's acceptance/rejection decision reflects the relative weight attributed to competing appraisals. For this overt behavioral decision, the (personal) appraisal of outcome favorability that drives (analytical) acceptance of goal-conducive outcome seems to be stronger than the (social) appraisal of outcome fairness, which may trigger covert (emotional) rejection of offers that are incompatible with justice norms. Our data show that the emotional reaction patterns provide a more fine-grained readout of the overall evaluation of the proposer's action, the underlying emotional dynamics of which may, in real life, strongly determine future interactions with specific partners. Further research on the relationship between emotion and behavior in economic games is needed to explore potential dissociations and long-term effects.

## Introduction

Justice is a major concern to individuals and societies [1]. Perceived injustice is a primary cause of negative emotions [2]–[4]. People are ready to incur costs in order to punish behaviors that are considered unfair because these behaviors deviate from a given justice norm [5], [6]. However, which norm is to be considered when evaluating the fairness of one's or others' actions depends on a variety of contextual [7] and individual factors [8]. Economic games have been widely used to study social behavior and norms, including justice. In these games, working for endowment (i.e., players have to earn the money that they play with) has a strong impact on what individuals perceive as fair [9], especially when endowments are heterogeneous [10], [11], suggesting that the perception and application of justice norms varies both within and across individuals. The present study investigated both intra- and inter-individual differences in the evaluation and application of justice norms in a modified ultimatum game (UG) involving the distribution of a jointly produced good with heterogeneous contributions.

Extensive research using the dictator game (DG) [12] and the UG [13], [14] shows that individuals take fairness concerns into account when making decisions about the distribution of a good (for reviews, see [15]–[18]). In classical versions of these games, anonymous individuals are paired. One member of the pair is randomly allocated the role of the dictator (DG) or proposer (UG), while the other is considered the recipient (DG) or responder (UG). The former is endowed with an amount of money that he or she can share with the latter. In the DG, the recipient cannot reject the offer, whereas in the UG, the responder has the power to reject an offer that he or she judges unfair, which results in higher offers in the UG compared with the DG. In both games, a significant proportion of individuals offer half of their initial endowment (others offer either nothing or little), suggesting that they consider equality as fair. However, many factors seem to affect the strength of the equality norm. For example, offers are smaller when the roles are allocated on the basis of merit compared with random choice, suggesting that the perceived fairness of role allocation procedures may reduce the strength of the equality norm. People may want to compensate a role allocation procedure that they judge (advantageously) unfair by applying an equality principle in order to be perceived as fair. Similarly, endowment origin (e.g., earned vs. unearned/windfall or randomly allocated) also results in more selfish behavior [19]–[23].

An even more complex situation arises when parties have contributed differentially to the production of a shared good [24]–[26] (see also [11] for similar findings in a public goods game). This scenario, which is an approximation of many real-life situations, introduces the equity norm, according to which a jointly produced good should be divided proportionally to each individual's input(s) as an alternative to the equality norm [27]. How should such a jointly produced resource be distributed among the different contributors? In a previous study, we showed that introducing a production phase, in which pairs of anonymous participants make different contributions to the production of a shared pie, strongly affected preferences for equality (i.e., 50–50 split) or equity (i.e., contribution-based split) justice principles during the distribution of this jointly produced good in a subsequent UG [28]. Responders who contributed less tended to favor the equality norm, whereas those who contributed more preferred the equity norm. However, only outright unfair offers that violated both justice principles were rejected. Proposers showed a similar egocentric bias in their offers, but they were able to anticipate the responders' reactions and adjusted their offers to avoid rejection, especially when an equitable split would have left the responder with very little and could thus be rejected. Overall, these studies show that egocentric biases not only affect judgments of fairness, but also drive the application of different justice norms in different situations. So far we have only considered preferences and strategic behavior. However, the emotional response to a proposed share is an important aspect of the game situation in itself and may well affect the decision and/or future behavior.

Fairness is an important criterion in the moral dimension of appraisal that underlies the elicitation and differentiation of emotion [29], [30]. The rejection of unfair offers in the UG is thought to be driven by the anger that these offers elicit [31], [32], an emotional reaction that has been associated with the appraisal of injustice or incompatibility with a justice norm [2], [33]. Note that disgust, a moral emotion associated with norm violation, has also been implicated in ultimatum rejections [34]. Following this reasoning, offers that are judged unfair should be rejected because they produce negative emotions, especially anger and disgust, which generally imply action tendencies of rejection and refusal (see [35]). In our previous study, although fairness ratings of equal and equitable offers varied inversely between production conditions, these offers were still accepted. This suggests that emotional reactions to unfair treatment might predispose the person to rejection (an impulsive action tendency) but that other considerations (such as monetary interest) and context factors may lead to a different behavioral choice. In this study, we examine the apparent incongruence between subjective fairness ratings and behavioral acceptance decisions by investigating the responders' emotional response patterns to offers. We hypothesize that the nature of the emotional response constitutes a more direct reflection of perceived norm compliance than the behavioral decision.

Besides the impact of contextual factors such as endowment origin and heterogeneity reviewed here, there are also important individual differences in the relative strength that individuals attribute to competing social preferences (e.g., self-interest vs. fairness) and to particular justice principles (e.g., equality vs. equity) [8], [36], [37]. For example, Cappelen et al. [8] describe three types of subjects who differ with respect to their behavior in a modified DG involving a production and distribution phase (see also Almas et al. [36] for slightly different terminology). Egalitarian individuals consider that all inequalities are unfair. In contrast, meritocratic individuals consider that efficiency or skills, efforts, and achievements, but not luck, can justify some inequalities. Finally, libertarians consider chance as an additional source of entitlement that can thus legitimate some inequalities. Thus, whereas egalitarians show a stable preference for equality across conditions, meritocratic and libertarians may weigh the multiple sources of entitlement differentially, resulting in different behaviors across both individuals and situations.

Another potentially important source of individual difference requiring further study concerns the (intraindividual) stability of individuals' preferences toward particular justice principles across different situations. In most studies, the DG and the UG are played in one-shot interactions, such that differences in behavior within and across conditions are likely to reflect different attitudes toward competing social preferences across individuals. Therefore, it is unclear whether the same individuals behave consistently according to a given social preference (self-interest vs. fairness) or according to a given justice norm (equality vs. equity). Egocentric biases suggest that individuals have stable social preferences and switch between justice principles in order to maximize their payoff in each and every situation (acting fairly in order to appear or to be perceived as fair simply being a means to avoid rejection) [38]. However, research also suggests that differences in the weight that individuals attribute to social preferences (e.g., individualism vs. fairness) may affect the weight they attribute to justice principles (e.g., equality vs. equity). The Social Value Orientation (SVO) questionnaire [39] measures the weights individuals attribute to their own (individual) and others' (social) outcomes. This questionnaire is frequently used to classify subjects according to their social preferences. Importantly, differences in SVO are likely to affect the stability of preferences for different justice principles. Individualists, for example, may show different preferences for equality and equity in different situations, depending on their self-interest (i.e. higher egocentric bias), whereas prosocials, in contrast, may show more stable preferences in order to maintain social fairness.

The purpose of this study was threefold: (1) We attempted to replicate the existence of an egocentric bias, as shown in Bediou et al. [28]. (2) We aimed to test the hypothesis that differences in social value orientation (SVO) may be associated with differences in egocentric biases. More specifically, we predicted that individualists would show a stronger egocentric bias than prosocials, resulting in more flexible preferences for equality and equity across situations (preferring equality or equity, depending on which norm is more self-advantageous), whereas pro-socials would show more stability (trading off between equality and equity in all situations). (3) We also attempted to extend the range of dependent variables to measure the effect of the different fairness conditions to analyze the detailed pattern of emotional responses to the treatment. Given that emotions are expected to reflect the complex appraisals a person makes of specific events and outcomes and to prepare appropriate action, we assumed that the emotional reactions would most faithfully reflect how participants evaluated the offers of the proposers. This would allow direct comparison of the emotional responses to the behavioral decisions.

## Methods

### Ethics statement

The study has been conducted according to the principles expressed in the Declaration of Helsinki. The design of the study was approved by the ethical committee of the Psychology Department of the University of Geneva, and all participants gave written informed consent before participating in the study.

### Participants

Thirty-six students (18 males, mean age 24 years old, range 18–33) were recruited through advertisement at the University of Geneva to participate simultaneously in a “game involving repeated anonymous computer-mediated interactions with other persons” in return for payment that would depend on their behavior during the experiment. Participants who asked for more information were told that they would be required to make a series of decisions, that their decisions would directly influence their payoff as well as the payoff of others, and that similarly, the decisions of others would affect their own as well as others' payoffs.

### Instruments

The instructions and questionnaires were similar to those used in Bediou et al. [28] and are briefly outlined in the procedure below. In this study, we used ratings of the intensity of the emotions that participants experienced as a consequence of the offer. To assess these emotional experiences, we used the Geneva Emotion Wheel (GEW), which allows us to obtain ratings on 20 emotion categories and their intensity (five levels) at the same time. The 20 emotional terms are organized according to two underlying dimensions of power (or control, potency) and valence (or pleasure, goal conduciveness), defining four different quadrants (see appendix): positive/high power (surprise, pride, joy, satisfaction, and pleasure), positive/low power (content, sympathy, admiration, relief, and compassion), negative/low power (sadness, guilt, regret, shame, and disappointment), and negative/high power (anxiety, disgust, contempt, envy, and anger). A detailed description of the instrument can be found in Scherer et al. [40]. Individual differences in social value orientation were assessed with a computerized version of SVO, which was administered to all subjects before they received further instructions about the task. Experimental constraints prevented us from administrating the SVO in advance, which could thus have produced carry-over effects on behavior during the experiment. Future studies should try to separate these two measures in time or at least counterbalance their order. We used the classic nine-item triple dominance measure [39], which classifies individuals as cooperators, individualists, or competitors if they make six of nine choices consistent with a certain SVO. Here we used the alternative scoring method, according to which an individual who makes six choices that are consistent with either an individualistic or competitive orientation would be classified (more broadly) as pro-self [41], [42]. Because 11 of the 12 pro-selfs were primarily classified as individualists, we refer to this group as individualists (N = 12), in opposition to prosocials (N = 18).

### Procedure

On arrival in the laboratory, participants were divided into two groups and directed to different rooms. Participants in each room were then told that they would participate in 28 independent interactions, each with a different and anonymous partner. Half of these partners would be students from the same university, all located in the other room (University of Geneva, 14 trials), whereas the other half would be students from a different university (University of Zürich, 14 trials). We then instructed the participants that each interaction would consist of two phases: a production phase and a distribution phase. In reality, there was no interaction between participants; all participants were always paired with a computer. The information about interactions with participants from Zurich was used as a cover story to allow multiple unique and anonymous interactions per participant, without reducing the believability of the interaction manipulation. Due to organizational constraints, we could not test two groups of 28 participants simultaneously, which would have been necessary to simulate the 28 interactions with a different anonymous “confederate” for each of our 36 actual participants. Subjects were informed that the outcomes of all interactions (i.e. the responders' decisions) would not be revealed until the end of the experiment and that they would be paid according to these interaction outcomes.

In the production phase, both players answered as many simple math calculations as possible in a limited amount of time in order to put more money into a shared pie. This phase allowed us to introduce heterogeneous contributions to the shared pie. In order to simulate the low and high contribution conditions, we used false feedback about the other (virtual) players' performance, which was based on the actual performance of the participant, plus or minus an additional random variation to increase realism of the online visual feedback. In half of the trials, the (virtual) other players' contribution was programmed to correspond to between 20% and 30% of the total shared pie (high contribution, 14 trials), whereas in the other half of the trials (low contribution, 14 trials), the virtual player was programmed to contribute between 70% and 80% of the of the total shared pie.

In the distribution phase, the money that had been produced by the participant and his or her presumed partner had to be distributed according to the rules of a UG, which were carefully explained to them. First, an ostensibly random allocation procedure determined who would be the proposer, and who would the responder, independently of their respective contributions. Proposers (12 trials) were asked to move a cursor between zero and the total amount of the pie in order to make an offer. On responder trials (N = 16) the computer was programmed to present, after a random delay (500–4500 ms), one of four different types of offers: equal offers (50% of the pie), equitable offers (based on contributions), unfair offers (10% of the pie), and hyperfair offers (90% of the pie). Each offer was presented only once in the low contribution conditions, and once in the high contribution, in a pseudo-randomized order. Responders were first asked to accept or reject each offer (in which case both players got nothing), and then to rate the fairness of each offer, as well as their emotional reaction to each offer. Thus, three types of measures were collected: participants' behavior (proposers' offers and responders' acceptance decision), their judgments (responders' ratings of the fairness of the offers using a 7-point scale), and their emotions (responders' self-reports on the GEW). Additional measures included participants' self-ratings of effort invested in the production phase and their satisfaction. Note that to avoid priming effects and to make the task easier, the order of the decision (accept/reject) and ratings (fairness and emotions) was kept constant both within and across subjects. Future studies may consider inverting this order to examine potential effects on a behavior/emotion discrepancy.

Before the game started, participants' attention was drawn to the following points that were written on a board and thus always visible: (i) All interactions are independent and anonymous. (ii) For each interaction, the allocation of roles is independent from the contributions. (iii) The responders' decisions will not be communicated to the proposers before the end of the experiment. (iv) You are playing for real money: The money that you will earn today depends on your decisions and the other players' decisions. After the experiment, subjects were debriefed individually and paid according to their interaction outcomes. Each participant was shown a summary table of all his/her interactions, including proposers' offers and responders' decisions. The outcomes of all these interactions was summed and added to a 5 CHF “show up” fee to determine participant payment. For proposer trials, we used a rule to realistically simulate responders' behavior in the ultimatum game, based on the literature and on the results of our previous study. All offers above 20% of the shared pie or above the participant's contribution were accepted. Offers below the contribution and below 20% of the shared pie were rejected. The average payment was CHF 72 (SD = 25, range 35–122). During debriefing, we explained the different experimental manipulations in detail and the reasons for them. Subjects were allowed to ask questions. None of the subjects suspected the reality of the interactions, and none of them reported negative feelings regarding their participation in the study.

## Results

### Preamble

Data from two subjects could not be retrieved because of computer failure after the experiment. We examined productions at the aggregate level as a manipulation check. The data from the proposers and the responders were then analyzed separately. First, we examined the impact of Contribution (low vs. high) on proposers' offers (6 trials in each contribution condition) in order to test the hypothesis that individualists show more flexibility in their selection of justice principles than prosocials do, as would be expected in the case of stronger egocentric bias. Second, we examined the impact of Contribution (low vs. high) and Offer (equality, equity, unfair, hyperfair) on responders separately for our three measures: acceptance decisions, fairness ratings, and emotions (2 trials in each condition).

### Manipulation check

We first checked whether the algorithm we used to create the high and low contributions showed the expected results and did not produce unwanted differences between conditions. As expected, participants produced a greater proportion of the pie in the high (72%) than in the low contribution condition (24%), *F*(1,32) = 7187.42, *p*<.001, but there was no difference between proposer and responder trials, all *Fs*<1. Of importance, participants produced significantly less than 50% of the pie in the low contribution condition, *t*(33) = 115.68, *p*<.001, and they produced significantly more than 50% of the pie in the high contribution condition, *t*(33) = 44.86, *p*<.001.

### Proposers' offers

#### Whole group analysis

**Figure 1:**
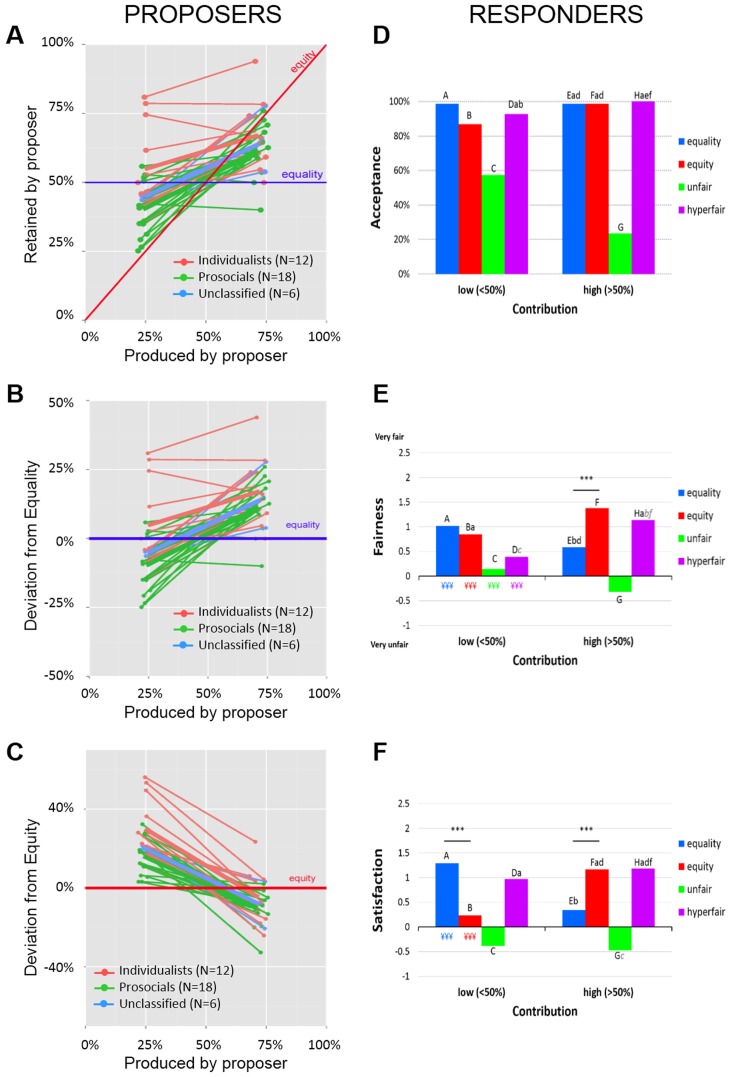
Proposers' and responders' behavior. (**A**) Proposers' offers. The equality and equity norms are shown in blue (horizontal) and red (oblique), respectively. (**B**) Deviation between proposers' offers and the equality norm (i.e., difference between a given offer and 50% of the pie) in the low and high contribution conditions. Each line corresponds to one individual and each value is the average of three trials. (**C**) Deviation between proposers' offers and the equity norm (i.e., difference between the percentage of the pie offered and the percentage produced) in the low and high contribution conditions. (**D**) Frequency of responders' acceptance decisions. (**E**) Fairness ratings (Z scores) on a 7-point Likert scale. (**F**) Satisfaction ratings (Z scores), extracted from the Geneva Emotion Wheel, including only five levels. Letters above the bars in Panels D to F indicate conditions that do not differ significantly from one another. Letters in gray italics indicate marginal effects. Asterics indicate significant differences between low and high contribution conditions.

We replicated the egocentric bias in the application of justice principles that we observed in our previous study [28] (Figure 1A). Participants retained more of the pie in the high contribution condition (64%±10) compared with the low contribution condition (46%±13), *t*(33) = 7.63, *p*<.001. Offers differed from equality in the high contribution condition, *t*(33) = 7.89, *p*<.001, but not in the low contribution condition, *t*(33) = 1.84, *p* = .075 (Figure 1B). Offers also differed from equity in both the high, *t*(33) = 4.61, *p*<.001, and the low contribution conditions, *t*(33) = 9.89, *p*<.001 (Figure 1C). In sum, in the low contribution condition, participants retained 50% of the pie (hence, significantly more than their actual contribution), whereas in the high contribution condition, participants retained more than 50% (though less than their actual contribution).

#### Individual differences

Despite the relatively low power afforded by our sample size for the analysis of individual differences, we decided to explore whether the egocentric bias observed at the aggregate level is predominantly produced by the individualists in the sample, given the importance of such a difference for further research. Prosocials' offers differed significantly from both equal and equitable splits in both contribution conditions. In the low contribution condition, prosocials retained significantly less than the equal split, *t*(17) = 4.54, *p*<.001, but significantly more than the equitable split, *t*(17) = 7.83, *p*<.001. In the high contribution condition, prosocials retained more than 50%, *t*(17) = 5.66, *p*<.001, but less than their production, *t*(17) = 5.50, *p*<.001. Individualists showed a different pattern than prosocials and a different pattern in the low and in the high contribution conditions. In the low contribution condition, individualists' offers differed from equity, *t*(11) = 6.79, *p*<.001, but not from equality, *t*(11) = 1.08, *p* = .30. This pattern was reversed in the high contribution condition, where individualists' offers differed from equality, *t*(11) = 4.99, *p*<.001, but not from equity, *t*(11) = 1.26, *p* = .23. Direct comparison of offers from individualists and prosocials in the low and high contribution conditions revealed significant group differences in the low contribution condition, *t*(32) = 3.28, *p* = .002, but not in the high contribution condition, *t*(32) = 1.34, *p* = .23.

### Responders' behavior

#### Acceptance decisions

As for proposers, again we replicated our previous findings, showing responders' tendency to reject only offers that at the same time are unfavorable and violate both the equality and the equity justice principles. A logistic regression with the factors Contribution and Offer showed a main effect of Offer, *F* = 64.77, *p*<.001, and a marginal effect of Contribution, *F* = 3.70, *p* = .055. More important, the Contribution x Offer interaction was also significant, *F* = 9.83, *p*<.001 (Figure 1D). Participants rejected unfair offers more often in the high (76%) than in the low contribution condition (43%), but there was no difference between low and high contribution conditions for equal, equitable, and hyperfair offers.

This suggests that there are fairly strong behavioral constraints to accept proposals even under clearly unfavorable conditions. What about the subjective evaluation of fairness?

#### Fairness ratings

Analysis of fairness and emotion ratings was based on raw data. Note that identical results were obtained with standardized (Z-transformed) ratings. Once again, we replicated our previous findings regarding fairness ratings [28], showing an egocentric bias in responders' fairness judgments (Figure 1E). A mixed-model ANOVA showed a main effect of Offer, *F*(3,96) = 34.26, *p*<.001, a marginal effect of Contribution, *F*(1,32) = 3.11, *p* = .087, as well as a significant Contribution x Offer interaction, *F*(3,96) = 30.78, *p*<.001. Equal and unfair offers were judged as fairer (or less unfair) in the low compared with the high contribution condition, whereas equitable and hyperfair offers showed the opposite pattern, being rated as fairer in the high contribution condition in comparison to the low contribution condition. Interestingly, in the low contribution condition equal and equitable offers were rated as being equally fair, *t*(33) = 5.24, *p*<.001, and both were considered fairer than hyperfair offers. This suggests that the saliency of the equality norm in the standard UG may be because the players distribute money that is not earned. In other words, adding a production phase in which participants first earn the money that they then have to distribute not only makes the task more ecologically valid, but it also induces feelings of entitlement, which may render the equity norm more salient than the equality norm.

#### Emotion ratings

**Figure 2:**
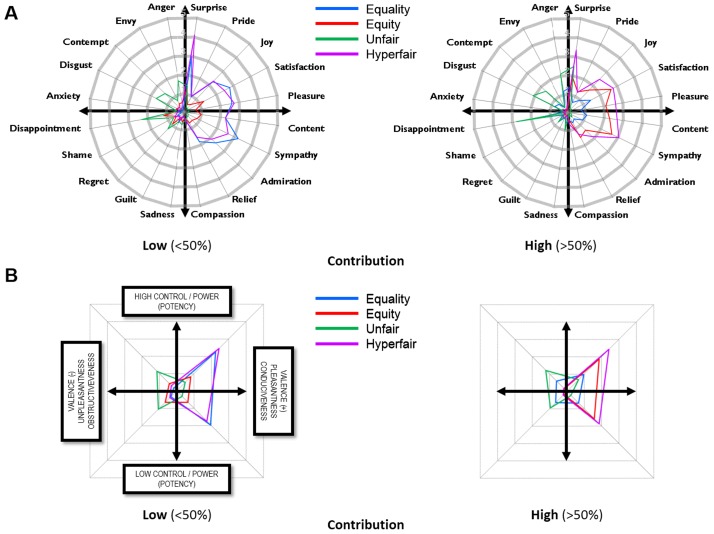
Responders' average emotional ratings of offers in low and high contribution conditions. The Geneva Emotion Wheel shows ratings (**A**) organized by emotion and (**B**) averaged across quadrants.

A multivariate repeated-measures ANOVA revealed a significant Contribution x Offer x Emotion interaction, *F*(57,1824) = 13.82, *p*<.001, suggesting that Contribution and Offer had a differential impact on different types of emotion (Figure 2A). Analysis on ratings averaged across quadrants gave very similar results. In the low contribution condition, equal offers elicited emotions that were similar to hyperfair offers, and equitable offers elicited emotions that were similar to unfair offers. This pattern was reversed in the high contribution condition, where equal offers were emotionally more similar to unfair offers and equitable offers more similar to hyperfair offers (Figure 2B).

In addition, we also replicated the egocentric bias for satisfaction ratings observed in our previous study [28] using a different measurement method. Again, there was a main effect of Offer, *F*(3,96) = 35.71, *p*<.001, and a Contribution x Offer interaction, *F*(3,96) = 20.88, *p*<.001 (Figure 1F). Satisfaction with equal offers was higher in the low compared with the high contribution condition, *t*(33) = 5.03, *p*<.001, whereas satisfaction with equitable offers was higher in the high compared with the low contribution condition, *t*(33) = 4.57, *p*<.001. Similarly unfair offers tended to be rated higher in the low than in the high contribution condition, *t*(33) = 4.57, *p*<.001, but there was no difference in satisfaction with hyperfair offers between the two contribution conditions. Comparing equal and equitable offers showed greater satisfaction with the former in the low contribution condition, *t*(33) = 4.85, *p*<.001, and greater satisfaction with the latter in the high contribution condition, *t*(33) = 4.18, *p*<.001.

#### Complex emotions

One of the specificities of the GEW in comparison to other self-report instruments is that subjects can report more than one emotion at the same time, with different intensities. Thus, we conducted a final exploratory analysis to investigate whether complex emotions, i.e., combinations of emotions from different quadrants of the GEW, would be identified and would differ across our experimental conditions. In order to improve data quality for this specific data-driven exploratory analysis, we first standardized the ratings (i.e., Z-transformed them) separately for each subject. Emotions for which the Z-scores were not normally distributed (skewness greater than 3, corresponding to the mean plus 1 SD) were excluded from further analysis (sadness, anxiety, and envy). An additional two emotions (shame and compassion) were excluded on the basis of kurtosis (greater than 10). A last group of four emotions (admiration, pride, sympathy, and relief) was also discarded for theoretical reasons. These emotions were not expected to arise in our experimental context (at least not in the responders), but were still included in order to maintain the coherence of the wheel in terms of dimensionality. A last set of two emotions was excluded on the basis of their low frequency of occurrence (regret and guilt). The remaining nine emotions were then submitted to further analysis (see Figure S1 and Table S1).

**Figure 3:**
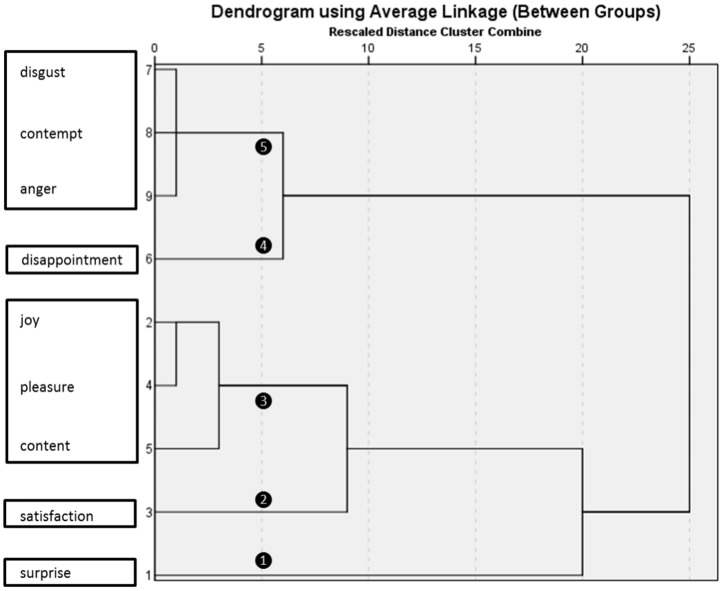
Hierarchical cluster analysis.

Second, Z-scores were submitted to hierarchical cluster analysis to identify the most frequent combinations of emotions across all conditions. Data-driven analysis identified six different clusters (Figure 3). To validate our result, we performed an exploratory principal component analysis (PCA) on the same data. Data-driven PCA revealed that a solution with two components explained 33% of the variance in the data. Additional PCAs with three, four, five, and six fixed factors were run to identify the best model; the corresponding solutions explained 46%, 63%, 78%, and 88% of the total variance, respectively (Table S2). Note that a model with five factors produced striking similarities between PCA components and hierarchical clusters.

**Figure 4:**
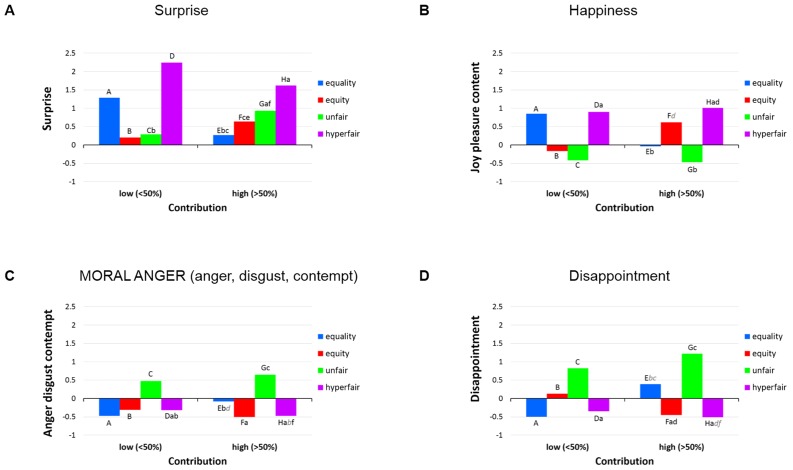
Effects of performance and offer on emotions extracted from hierarchical cluster and PCA analyses. Conditions that do not differ significantly from one another are marked with the same letters.

Third, we computed composite scores for the following emotion combinations, by simply averaging the Z-scores of each and every emotion that belonged to a given cluster (or component): disgust, anger, and contempt (MORAL ANGER) and joy, pleasure, and content (HAPPINESS). These complex emotions, as well as remaining individual emotions (disappointment, satisfaction, and surprise), were submitted to repeated measures ANOVAs to examine the impact of performance and offer on these simple and complex emotions (Table 1 and Figure 4). Significant interactions were followed up by post hoc comparisons using Tukey's honest significant difference test.

**Table 1:** Repeated-measures ANOVA results on emotion ratings.

	Performance		Offer		Performance x Offer	
	F	p	F	p	F	p
Surprise	1.17	.29	**27.52**	**<.001**	**12.62**	**<.001**
Satisfaction	**4.13**	**.05**	**4.19**	**.008**	**7.42**	**<.001**
HAPPINESS (joy, pleasure, content)	.04	.84	**45.61**	**<.001**	**30.157**	**<.001**
Disappointment	2.52	.122	**51.34**	**<.001**	**13.05**	**<.001**
MORAL ANGER (anger, disgust, contempt)	.16	.70	**48.97**	**<.001**	**5.86**	**<.001**

*Note.* Capitalized terms refer to composite scales (terms of underlying emotions in parentheses).

In the following description, we interpret the major patterns of significant findings that emerge from a systematic comparison of the panels in Figure 4. Most strikingly, panels C and D show an overwhelming effect of Unfairness on both Disappointment and Moral anger, with very little variation for the other offer conditions. While the means seem to imply that this effect is stronger in the high contribution condition, there is no significant difference in the post hoc tests. This seems to suggest that the strong violation of the pervasive fairness norm produces an unequivocal emotional response. This is particularly underlined by our composite variable Moral anger, which also contains disgust and contempt. Judging from the data, one is tempted to assume that Disappointment might also have a moral connotation here, i.e., possible disappointment about the integrity or norm compliance of the proposer.

Panel A shows a very strong surprise effect for hyperfair offers (in particular for low contribution conditions). This is not surprising, as such offers tend to be extremely rare in real life, thus violating very strong expectations in the present case. Of interest is also the rather strong surprise reaction of low performing participants in the equality condition, probably reflecting that such a pattern of distribution is somewhat unexpected. One might expect that high performers should be equally or even more surprised, given their sense of entitlement, but this is not the case. They are in fact much less surprised than the low performers. In contrast, they are more surprised about unfairness than the low performers. On the whole, the pattern for surprise seems to suggest that low performance/contribution generally lowers the expectation for a favorable outcome.

As to Panel B, as one might expect, high performers are happier with equitable distributions, whereas low performers are happier with equality. Both the low and the high contribution groups are unhappy with unfair distributions (mirroring moral anger and disappointment). Both groups are not only surprised, but also quite happy with hyperfair solutions, which is probably explained by the fact that the payoff is higher than expected, relegating equity issues to a lower level of importance.

As in the case of the proposers' offers we conducted an exploratory analysis of individual differences in responders' decisions and emotions. Again, as these analyses involve only a small subsample of the participants, they are rather under-powered and should be interpreted with great caution. A first analysis conducted on data collapsed across all offer types in the low and high contribution conditions showed no significant difference between prosocials and individualists in either the frequency of acceptance decisions or the overall emotional ratings (collapsed across the 20 emotion categories). A second analysis focused on the unfair offers, which gave the highest rejection rate and thus provide a window into the determinants of rejection. In order to increase statistical power, data from the low and high contribution conditions were collapsed. The main goal of this analysis was to address (1) whether individualists and prosocials differ in their emotional reactions to these offers, and (2) whether these emotional reactions (in particular MORAL ANGER) predict their decisions or not. Although there were no significant differences between prosocials and individualists in terms of behavior (acceptance rates: *t*(28) <1, *p* = 0.58) and emotions (levels of moral anger: *t*(28) = 1.1, *p* = 0.28), a logistic regression analysis on acceptance decisions, with moral anger and SVO as continuous and categorical predictors, respectively, revealed a significant interaction, Wald chi-square = 3.68, *p* = .055. Increased moral anger predicted lower acceptance rates in individualists (R^2^ = 0.36, *p* = .055), but not in prosocials (R^2^ = 0.02, *p* = .546). Although the small sample size calls for cautious interpretation of this exploratory analysis, this suggests that prosocials tended to better regulate their emotionally driven rejection of these very unfair offers (Figure S2).

## Discussion

The present study had three main goals. First, we replicated the results of our first study, showing egocentric biases in the perception and application of justice principles in a UG with heterogeneous contributions to a shared pie. In the standard UG, participants distribute money provided by the experimenter. In this situation, there is no perceived difference in entitlement. In consequence, the equality norm may seem to be the most salient and appropriate. In contrast, the equity norm appears to be stronger and more salient than the equality norm when earned money is distributed. This extends the idea that working for endowment strongly affects behavior in economic games [9] by showing that it also affects preferences for equal versus equitable outcomes. Second, we investigated the impact of individual differences in SVO on proposers' offers. We report these exploratory analyses despite the fact that they are somewhat under-powered as we believe that even preliminary indices for the importance of such individual differences may provide useful information for work in this area. We found that individualists tended to be more flexible in their application of justice norms, consistent with having a greater egocentric bias than prosocials. When their contribution to the shared pie was low, individualists mostly offered equal splits, whereas they offered equitable splits when their contribution was high. This suggests that they may have weighted these two norms differentially (and egocentrically) in order to maximize their gain in each and every situation. In contrast, offers from prosocial proposers always reflected a compromise between the two norms, which suggests more stable preferences [43]. This type of behavior is consistent with the predictions from bargaining theories of coalition formation (see review in [44]). Although individualists played with justice norms, it is noteworthy that their offers rarely violated both norms, suggesting that they were able to anticipate that the responders may reject offers violating both norms. This may be viewed as a more “strategic” behavior based on mentalizing abilities (e.g., perspective taking), as opposed to the more “empathic” behavior of prosocials. Further research is needed to better understand the origin of these differences, for example, examining whether they arise from different motivations or through different cognitions or emotions [45].

Our third goal was to investigate the emotional correlates of the responders' egocentric bias in the perception of the equality and equity justice norms in this same game. Although responders showed an egocentric bias in their judgments of the fairness of offers, they accepted all offers that could be justified by either the equality or the equity norm, and they rejected outright only those offers that violated both principles, a result that replicated our previous findings [28]. Responders showed a similar egocentric bias in their emotions, much more in line with their fairness judgments than with their acceptance decisions. On the one hand, this result is consistent with the idea that the appraisal of (in)justice is an important determinant of emotions, particularly anger. On the other hand, the fact that responders were asked to report their emotions may have helped to reduce the intensity and impact of their emotions [46], resulting in lower levels of negative emotions (see Figure 2B) and a lower frequency of decisions to reject the offers [47].

The present study contributes to the existing literature on egocentric biases and justice/fairness norms in at least three ways. First, we found an egocentric bias in responders' evaluations of the perceived fairness of equal and equitable offers, depending on their contribution to the joint production, replicating the results of our previous study [28]. Second, we showed that these egocentric biases are also present at an emotional but not at a behavioral level. Equal and equitable offers that were disadvantageous (e.g., equitable offer in low contribution condition and equal offer in high contribution condition), were rated as less fair and associated with emotions similar to those associated with unfair offers, but these offers were still accepted. One could thus speculate that a form of emotion regulation has taken place in order for responders to accept offers that are economically disadvantageous but comply with either the equality or the equity justice principle. In order to maximize one's own gain, a responder should refrain from his or her emotional impulse to punish a greedy but not-so-unfair proposer for a more rational decision to accept the proposed offer. To do so, it may be useful to emphasize the individual (economic) aspect of the game while neglecting the social (fairness) dimension, which could be achieved, for example, by reappraising the offer as arising from a nonintentional agent (e.g., a computer). Third, additional exploratory analyses suggest that prosocials might have more stable preferences and more consistent behavior across conditions than individualists [43]. Several possible explanations can be adduced for these findings: One possibility is that individualists and prosocials appraise the same offers differently [48] and thus anticipate different reactions in the responders, leading to different behavior. Another possibility is that individualists and prosocials appraise the offers similarly, but weight this information differently. For example, they may use different degrees of perspective taking and empathy (i.e., anticipating the responder's emotional reaction) to guide their behavior. Responders' behavior and ratings should thus be particularly informative in that respect. Consistent with the second alternative, exploratory analyses revealed no significant difference between prosocials and individualists in terms of acceptance or emotional reactions to unfair offers. However, moral anger predicted rejection in individualists only, not in prosocials, suggesting that the latter group regulated their emotions. Once again, we underline that these individual difference results need replication to examine their stability and should thus be interpreted cautiously given the small sample size involved in these analyses.

Analysis of responders' emotions not only sheds lights on the determinants of their acceptance/rejection decisions, which are thought to lie in their emotional reactions to these offers, but also provides a wealth of information about the appraisal dimensions on which these offers may be evaluated. In the UG, a responder is likely to appraise a proposer's offer on at least two dimensions: goal conduciveness (how good is the offer?) and norm compatibility (how fair is the offer?), which can be seen as two types of valence that may interact and even conflict [49]. Both judgments involve the comparison of the offer received with an expected offer. Goal conduciveness may be assessed by comparing the amount offered with the amount expected, which may depend on the individual's estimated production (in absolute terms), or contribution to the joint production (in relative terms). In contrast, norm compatibility may be derived from the comparison of the proposed distribution with an expected (i.e., fair) distribution based on the responder's preferred norm (e.g., equality or equity). In addition to rendering the task more ecological, our joint-production manipulation may have affected the subjects' norm-compatibility expectations. Our results suggest that the responder may expect different norms to be applied in different contexts: equality in case of low contribution and equity in case of high contribution. In this study, emotion ratings were always collected after a decision was made. These ratings were affected by our experimental conditions in the expected directions. We cannot exclude that some form of regulation had already taken place when the participant accepted or rejected the offer. Although this would account for the lower levels of negative compared with positive emotions (compare the left and right sides of the wheel in Figure 2), a replication study assessing emotions first (i.e. before accept/reject decisions) would be useful to confirm these effects and their interpretation. More generally, our results encourage a more systematic assessment of the relationship between justice-related emotions and behavioral decisions, including an examination of emotion regulation mechanisms and the differential role of social norms for emotions and behavior.

Moreover, goal conduciveness and norm compatibility are likely to interact. Favorable outcomes are rated higher on both distributive and procedural fairness, and people report higher satisfaction with outcomes when they consider that either the outcomes themselves or the procedure that arrived at these outcomes was fair [19]–[23] (for a review, see [50]). In addition, there is an asymmetry in both satisfaction and fairness judgments, with satisfaction and fairness being lower for disadvantageous inequality in comparison to advantageous inequality [3], [28], [51], [52]. In a UG with heterogeneous contribution to a jointly produced good, a responder may expect an equal or equitable split, depending on what is more advantageous in the situation at hand. The responder may thus be torn between what he or she wants to do (goal conduciveness, selfish expectation favors acceptance) and what he or she should do on the basis of a justice norm (norm compatibility, fairness expectation favors rejection). In the UG, an outcome that is worse than expected based on one's preferred justice principle is more likely to be perceived as unfair (justice appraisal). Whether this offer will be rejected depends on additional factors, such as whether the offer is also worse (in magnitude) than what was expected based on one's initial contribution (favorability appraisal).

The motivations driving norm-compliance behavior remain a puzzle and competing theories have developed. One group of theories proposes that individuals have a preference for fairness and are thus intrinsically motivated to act fairly [53]. Another group proposes that fairness is driven by self-interest, either because people expect reciprocity [54], or because they want to maintain their social image and be perceived as fair [55], as this may turn out to be advantageous in the future. In a UG with heterogeneous contribution to the to-be-shared pie, proposers and responders may have different expectations regarding which of the equality or equity norms is more appropriate (or advantageous). Our results suggest that both groups of theories (social preference vs. strategic self-interest) may be valid, but present in different individuals. Prosocials have more stable preferences for fairness irrespective of the most advantageous norm, whereas individualists try to maximize their personal gain while maintaining a positive social image as a fair person (i.e., they conform to an existing norm: the most advantageous one). Overall, the present study highlights the importance of both inter- and intraindividual differences in the perception and application of justice principles, consistent with the idea that justice is context dependent [56]. Importantly, the exploratory nature of the individual difference analyses reported here call for further research in that direction, given their theoretical importance for the understanding of the underlying mechanisms.

In conclusion, we propose that the emotional reactions, reflected in subjective experience, possibly provide a more veridical assessment of a person's reaction to a distribution offer than overt acceptance responses (despite the potential biases involved in self-report measures). Emotions are reactions to the appraised consequences of an event, like a distribution offer, and they prepare an appropriate action tendency [57], but they must not be confused with overt action or behavior that is determined by many additional factors, both internal (motivation) and external, such as social constraints [58], [59]. It could be argued that what counts is the overt behavior, the decision taken to reject or accept, but that is true only within the experimental paradigm of economic games in which one's interaction partner is mostly anonymous (or a computer) and in which short time frames and few exchanges are involved. In reality, we usually know our partners and exchanges are often not limited to single, short episodes. Under these conditions, the emotional reaction to the perceived fairness or unfairness of the other may actually be much more influential than the immediate behavioral reaction to a single offer, as these emotional reactions are likely to affect trust and liking toward the other and thus strongly determine future interactions, decisions, and outcomes. Thus, if we want to better understand the effect of fairness perceptions in social and economic exchanges, we probably need to devote greater attention to the underlying emotional dynamics than has been the case to date.

## Supporting Information

Figure S1 **The Geneva Emotion Wheel (version used in the present experiment).** The emotions that were excluded from the analysis are shown with a dashed contour for ease of reference.
(TIF)

Figure S2 **Individual differences in responders' decisions and emotions in response to unfair offers (collapsed across both contribution conditions).** **(A)** Moral anger ratings and acceptance rates for unfair offers. **(B)** Scatter plots of the relationship between moral anger and acceptance rates.
(TIF)

Table S1 **Frequencies of occurrence of each emotion.**
(DOCX)

Table S2 **Principal component analysis.**
(DOCX)
