# Supplementary figures and images for: Egocentric Fairness Perception: Emotional Reactions and Individual Differences in Overt Responses
Source: PLoS One. 2014 Feb 28;9(2):e88432. doi: 10.1371/journal.pone.0088432 (PMC3938425; doi:10.1371/journal.pone.0088432)

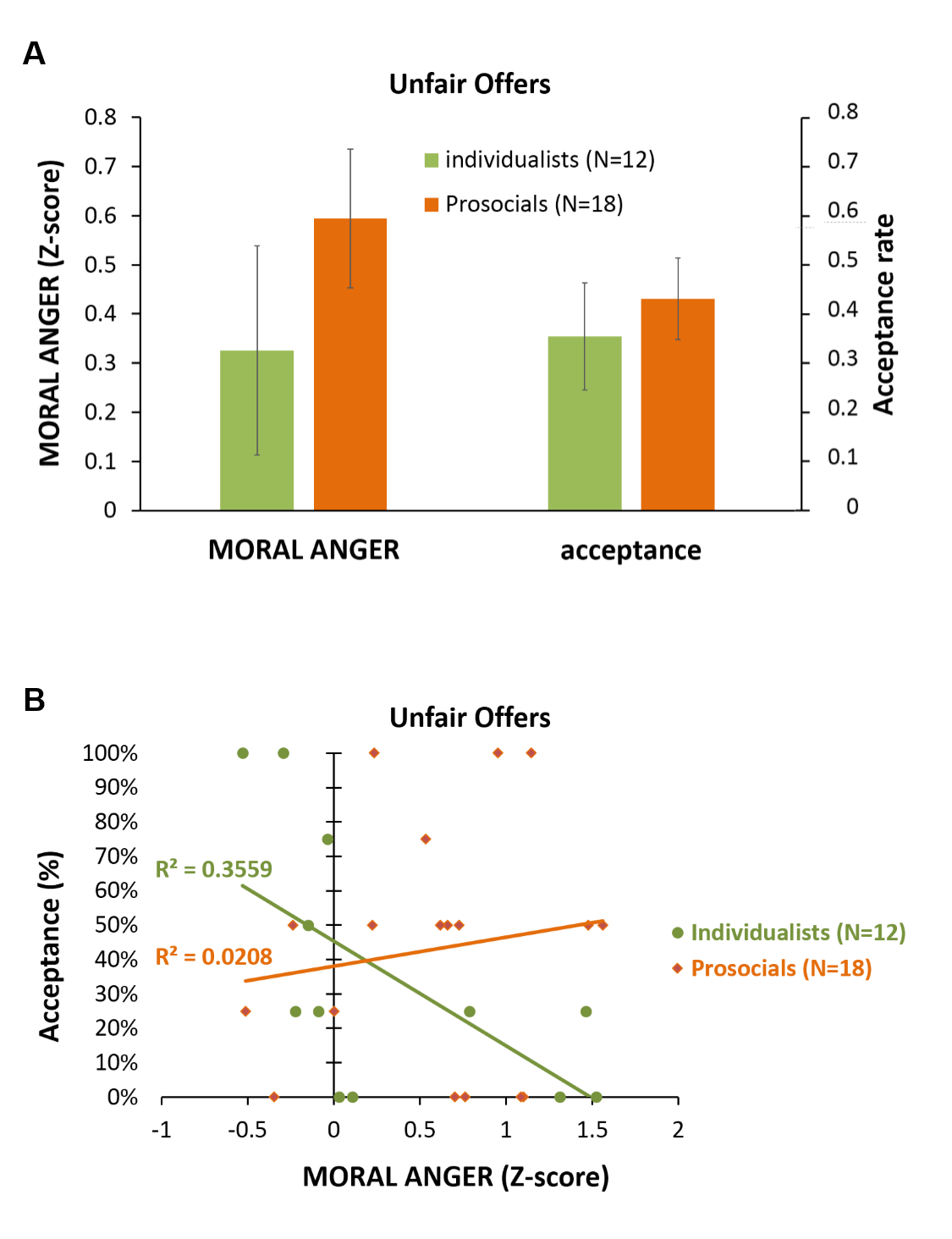

Supplement: Figure S2 — Individual differences in responders' decisions and emotions in response to unfair offers (collapsed across both contribution conditions). (A) Moral anger ratings and acceptance rates for unfair offers. (B) Scatter plots of the relationship between moral anger and acceptance rates. (TIF) [file pone.0088432.s002.tif]
