# Supplementary material for: Egocentric Fairness Perception: Emotional Reactions and Individual Differences in Overt Responses
Source: PLoS One. 2014 Feb 28;9(2):e88432. doi: 10.1371/journal.pone.0088432 (PMC3938425; doi:10.1371/journal.pone.0088432)
Supplement: Table S1 — Frequencies of occurrence of each emotion. (DOCX) [file pone.0088432.s003.docx]

**Table S1.** Frequencies of occurrence of each emotion.

| *Anxiety* | *76* |
| --- | --- |
| *Envy* | *78* |
| *Shame* | *84* |
| *Guilt* | *93* |
| *Compassion* | *98* |
| *Sadness* | *105* |
| *Regret* | *114* |
| Contempt | 119 |
| Disgust | 135 |
| *Pride* | *137* |
| Anger | 145 |
| *Relief* | *167* |
| *Admiration* | *173* |
| Disappointment | 179 |
| Joy | 196 |
| Pleasure | 225 |
| Content | 225 |
| *Sympathy* | *242* |
| Satisfaction | 267 |
| Surprise | 318 |

*Note.* Emotions that were discarded from the exploratory analysis are shown in italics.
