# Supplementary material for: Egocentric Fairness Perception: Emotional Reactions and Individual Differences in Overt Responses
Source: PLoS One. 2014 Feb 28;9(2):e88432. doi: 10.1371/journal.pone.0088432 (PMC3938425; doi:10.1371/journal.pone.0088432)
Supplement: Table S2 — Principal component analysis. (DOCX) [file pone.0088432.s004.docx]

**Table S2.** Principal component analysis.

1. Test of different models.

| Component | Initial Eigenvalues^a^ | | | Extraction Sums of Squared Loadings | | | Rotation Sums of Squared Loadings | | |
| --- | --- | --- | --- | --- | --- | --- | --- | --- | --- |
|  | Total | % of Variance | Cumulative % | Total | % of Variance | Cumulative % | Total | % of Variance | Cumulative % |
| 1 | 4.622 | 40.791 | 40.791 | 4.622 | 40.791 | 40.791 | 1.939 | 17.114 | 17.114 |
| 2 | 1.845 | 16.283 | 57.074 | 1.845 | 16.283 | 57.074 | 1.823 | 16.085 | 33.200 |
| 3 | 1.238 | 10.923 | 67.997 | 1.238 | 10.923 | 67.997 | 1.431 | 12.628 | 45.828 |
| 4 | .901 | 7.956 | 75.953 | .901 | 7.956 | 75.953 | 1.956 | 17.260 | 63.088 |
| **5** | **.769** | **6.785** | **82.737** | **.769** | **6.785** | **82.737** | **1.636** | **14.442** | **77.531** |
| 6 | .623 | 5.497 | 88.234 | .623 | 5.497 | 88.234 | 1.213 | 10.704 | 88.234 |
| 7 | .475 | 4.195 | 92.429 |  |  |  |  |  |  |
| 8 | .439 | 3.875 | 96.305 |  |  |  |  |  |  |
| 9 | .419 | 3.695 | 100.000 |  |  |  |  |  |  |

1. Model with five factors: rotated components matrix.

| Emotion | Cluster | Component | | | | |
| --- | --- | --- | --- | --- | --- | --- |
|  |  | 1 | 2 | 3 | 4 | 5 |
| Pleasure | 5 | **.920** | -.186 | .214 | -.141 | .146 |
| Joy | 5 | **.888** | -.184 |  |  | .124 |
| Content | 5 | **.851** | -.217 |  | -.203 | .223 |
| Disgust | 3 | -.138 | **.777** |  | .193 |  |
| Anger | 3 | -.200 | **.773** |  |  | -.116 |
| Contempt | 3 | -.119 | **.668** |  |  |  |
| Surprise | 1 | .280 |  | **1.364** |  |  |
| Disappointment | 4 | -.261 | .299 |  | **1.029** | -.143 |
| Satisfaction | 2 | .417 | -.232 |  | -.168 | **1.208** |

*Note.* Factors with absolute loadings (i.e., weights) greater than .5 are highlighted in bold.
